# Effects of whole-body electromyostimulation as a time-efficient intervention on myokine profile and muscular performance in overweight adults: a randomized controlled study

**DOI:** 10.3389/fspor.2026.1761170

**Published:** 2026-05-19

**Authors:** Fatemeh Mahboobi Semnan, Mohammad Bolbol Anbaran, Mahdi Bayati, Sadegh Amani-Shalamzari, Lara Carneiro

**Affiliations:** 1 Department of Exercise Physiology, Faculty of Physical Education and Sports Science, Kharazmi University, Tehran, Iran; 2 Department of Exercise Physiology, Sport Sciences Research Institute, Tehran, Iran; 3 Physical Education Department, College of Education, United Arab Emirates University, Abu Dhabi, United Arab Emirates

**Keywords:** exerkine, fitness, hypertrophy, obesity, resistance training

## Abstract

**Introduction:**

This study compared the efficacy of whole-body electromyostimulation (WB-EMS) vs. traditional resistance training (RT) in overweight adults.

**Methods:**

Forty overweight adults were randomized to WB-EMS (*n* = 14), RT (*n* = 14), or a control group (*n* = 12). Five participants withdrew during the intervention; consequently, and 35 participants completed the study and were included in the final analysis: WB-EMS (*n* = 12; age = 30 years, BMI = 29.12 kg/m^2^; 20-min sessions, 30 Hz, 350*μ*s), RT (*n* = 12; age = 30 years; BMI = 29.14 kg/m^2^; 3 × 10 reps at 55%-70% 1RM), or control (*n* = 11, age = 29 years; BMI = 29.17 kg/m^2^). The intervention lasted 8 weeks with biweekly training sessions. Outcome measures included muscular performance tests, ultrasonographic muscle thickness measurements, and serum biomarkers (IL-15, myostatin, and follistatin). Between-group differences were analyzed using ANCOVA with baseline values as covariates.

**Results:**

Findings demonstrated significantly greater improvements in selected performance and biomarker outcomes in the WB-EMS group compared to other groups. Performance tests revealed significant enhancements in sit-ups (*η*_p_^2^ = 0.35), push-ups (*η*_p_^2^ = 0.54), and strength measures (*η*_p_^2^ = 0.46) compared to RT and control groups. Biomarker analysis showed WB-EMS induced a 15.88% increase in IL-15 vs. 8.27% with RT and −4.92% in controls (*p* < 0.001). Myostatin decreased by 21.22% (WB-EMS) vs. 10.84% (RT), while increasing 5.08% in controls (*p* < 0.001). Follistatin levels rose by 17.96% (WB-EMS) and 8.92% (RT), with minimal change in controls (0.03%, *p* < 0.001). All between-group differences were statistically significant (*p* = 0.016-0.039) with large effect sizes (*η*_p_^2^ = 0.35-0.63).

**Conclusions:**

Twenty-minute WB-EMS sessions twice weekly elicit significantly greater improvements in muscular performance and favorable myokine profiles compared to conventional RT in overweight individuals. These findings suggest that WB-EMS may represent a time-efficient alternative for improving metabolic-muscular health in time-constrained populations.

## 1 Introduction

The rise in sedentary lifestyles, coupled with excessive caloric intake, has contributed to a global increase in obesity rates (1). Beyond its role as an energy reservoir, adipose tissue can modulate the function of other tissues through the release of adipokines. Notably, obesity also impairs skeletal muscle endocrine function, altering the secretion of critical myokines. Especially, obesity is characterized by an imbalance in myokine regulation, notably reflected in elevated serum myostatin (MSTN) (a muscle growth inhibitor) (2), elevated/unchanged follistatin (a myostatin antagonist) (3, 4), alongside reduced interleukin-15 (IL-15) levels (5, 6), collectively resulting in skeletal muscle atrophy, adipose tissue hypertrophy, persistent low-grade inflammation, and metabolic disturbances. Hence, obesity results in a reduced follistatin-to-myostatin (F:M) ratio, a critical regulator of skeletal muscle growth (7). These perturbations contribute to metabolic dysregulation (e.g., insulin resistance), elevated cardiovascular risk, and muscle atrophy—key hallmarks of obesity-related morbidity. Given these systemic effects, interventions targeting myokine rebalancing, including structured exercise, have emerged as promising strategies to mitigate obesity-associated complications (8).

Overweight and obesity are associated with reduced cardiorespiratory fitness, increased joint loading, chronic low-grade inflammation, and impaired skeletal muscle function, all of which may limit tolerance to conventional high-volume resistance training. Common barriers in this population include musculoskeletal discomfort, fatigue, low exercise adherence, and time constraints. Therefore, alternative training modalities that reduce mechanical strain while preserving metabolic and neuromuscular stimulation may offer practical advantages in clinical and community settings.

Exercise training represents a cornerstone of obesity management, with improvements in body composition, insulin sensitivity, and cardiometabolic risk partly mediated by contraction-induced exerkines (9, 10). Regular exercise has been shown to increase IL-15 (6, 11, 12) and reduce MSTN concentrations (13), thereby favorably modulating the F:M ratio, an important regulator of skeletal muscle mass and metabolic function (7). At the molecular level, resistance exercise stimulates Akt/mTOR signaling while attenuating myostatin–SMAD2/3 activity, promoting muscle protein synthesis and counteracting obesity-associated anabolic resistance (14).‏ Beyond local muscular adaptations, myokines such as IL-15 facilitate muscle–adipose tissue crosstalk by modulating lipid metabolism and inflammatory signaling, underscoring the systemic endocrine role of skeletal muscle in obesity (15). Despite these well-established molecular benefits, adherence to conventional exercise regimens remains low due to time constraints and sedentary behavior, especially among the overweight adult population. This compliance gap has spurred interest in time-efficient alternatives like whole-body electrical muscle stimulation (WB-EMS), which may mimic the metabolic and myokine-modulating effects of voluntary exercise (16). EMS, by eliciting muscle contractions via external electrical impulses, may activate an intracellular pathway associated with metabolic adaptation.

WB-EMS has emerged as an innovative training modality that provides a time-efficient, joint-friendly, and highly adaptable exercise approach. By employing electrical impulses delivered via electrodes embedded in a specialized suit, WB-EMS elicits synchronous activation of multiple major muscle groups, with adjustable intensity tailored to specific anatomical regions (17). Unlike voluntary resistance exercise, which follows the physiological size principle of motor unit recruitment, electrically evoked contractions involve a more synchronous and spatially fixed activation pattern, potentially altering mechanical loading and metabolic stress (18). The resistance training (RT) and WB-EMS have the ability to activate intracellular pathways that are related to metabolic, oxidative, and calcium-mediated adaptations, but the magnitude and timing of these responses may be influenced by differences in recruitment patterns and contraction synchrony (19, 20).‏ Despite these differences, emerging evidence indicates that WB-EMS can induce improvements in muscle mass, strength, and body composition comparable to those observed following high-intensity RT (21). When discussing equivalence between WB-EMS and conventional RT, it is important to distinguish between functional outcomes and potential differences in neuromuscular recruitment, mechanical tension, and endocrine responses. A single 20-minute WB-EMS session per week has been reported to achieve similar functional outcomes to 60 min of conventional RT, highlighting its potential time-efficiency (22). WB-EMS has demonstrated efficacy in preserving lean body mass and enhancing muscular strength, particularly in sedentary individuals (23–26). Additionally, Vadala et al. (2025) reported significant increases in serum irisin one hour post-exercise compared to aerobic exercise alone (27). These findings indicate that WB-EMS may provide a feasible option for improving muscle strength and body composition in populations with limited time for structured exercise.

Given the prevailing socioeconomic challenges and limited access to structured exercise programs, there is a growing interest in time-efficient training methods such as WB-EMS that could maximize physiological benefits within minimal timeframes. This study specifically examines whether 16 sessions of 20-minute WB-EMS can induce meaningful changes in body composition and serum myokine levels (IL-15, follistatin, and MSTN) compared to traditional RT of equal duration and a non-exercising control group. We hypothesized that both WB-EMS and RT would lead to significant improvements in body fat percentage, body mass index (BMI), muscle thickness of the rectus abdominis and biceps brachii, physical performance measures (muscular endurance and strength), and circulating myokine levels (IL-15, follistatin, and MSTN, F:M ratio) compared with the control group. In addition, we hypothesized that potential differences between the two active interventions would be evaluated without *a priori* assumptions regarding the superiority of either modality. No directional superiority hypothesis was formulated between WB-EMS and RT due to heterogeneity in stimulus characteristics and limited head-to-head comparative evidence in overweight/obese populations. The findings could provide preliminary evidence on the potential utility of WB-EMS and RT as practical options for populations with limited time for structured exercise.

## 2 Materials and methods

### 2.1 Study design

This randomized, single-blind trial included This randomized, single-blind trial included forty overweight and obese adults who were randomly assigned to one control group or one of two parallel experimental groups. Laboratory personnel responsible for outcome assessments were blinded to group allocation. The primary hypothesis of this randomized controlled trial was that both WB-EMS and RT would produce statistically significant changes from baseline to post-intervention in muscle thickness (rectus abdominis and biceps brachii), body fat percentage, muscular endurance and strength performance, and circulating myokines (interleukin-15, myostatin, follistatin, and the follistatin-to-myostatin ratio) compared with the control group. A secondary hypothesis was that between-group differences would be observed between WB-EMS and RT; however, no *a priori* assumption regarding superiority of either intervention was made. All participants provided written informed consent after receiving comprehensive information regarding the study procedures, potential risks, and anticipated benefits. The study was conducted in accordance with the Declaration of Helsinki and was approved by the Ethics Committee of the Sport Sciences Research Institute of Iran (protocol code: IR.SSRC.REC.1402.250; approval date: 2023-11-21). The intervention commenced on December 1, 2023, and was completed after eight weeks.

### 2.2 Randomization and blinding

Participants were randomly allocated to one of three groups: whole-body electromyostimulation (WB-EMS, *n* = 14), resistance training (RT, *n* = 14), or control (C, *n* = 12). Randomization was performed using a computer-generated block randomization procedure with a block size of six to ensure balanced group allocation across the three study arms. The random allocation sequence was generated and implemented by an independent third party who was not involved in participant recruitment, assessment, or intervention delivery. Allocation was concealed until participants completed baseline assessments. The study followed a single-blind design, in which laboratory personnel conducting outcome measurements were blinded to group assignment throughout the study period. Prior to enrollment, all participants were screened using a standardized contraindications checklist for WB-EMS, including assessment of cardiovascular disease, implanted electronic devices (e.g., pacemakers), epilepsy, pregnancy, acute infections, and other established contraindications. Eligibility screening was conducted using a standardized health screening questionnaire completed by all potential participants. The completed questionnaires were reviewed by the principal investigator, who verified eligibility based on the predefined inclusion and exclusion criteria prior to enrollment. The screening procedure was conducted in accordance with published safety recommendations for WB-EMS application (17). Only participants who met all eligibility requirements were enrolled and subsequently randomized.

### 2.3 Participants

**Figure 1:**
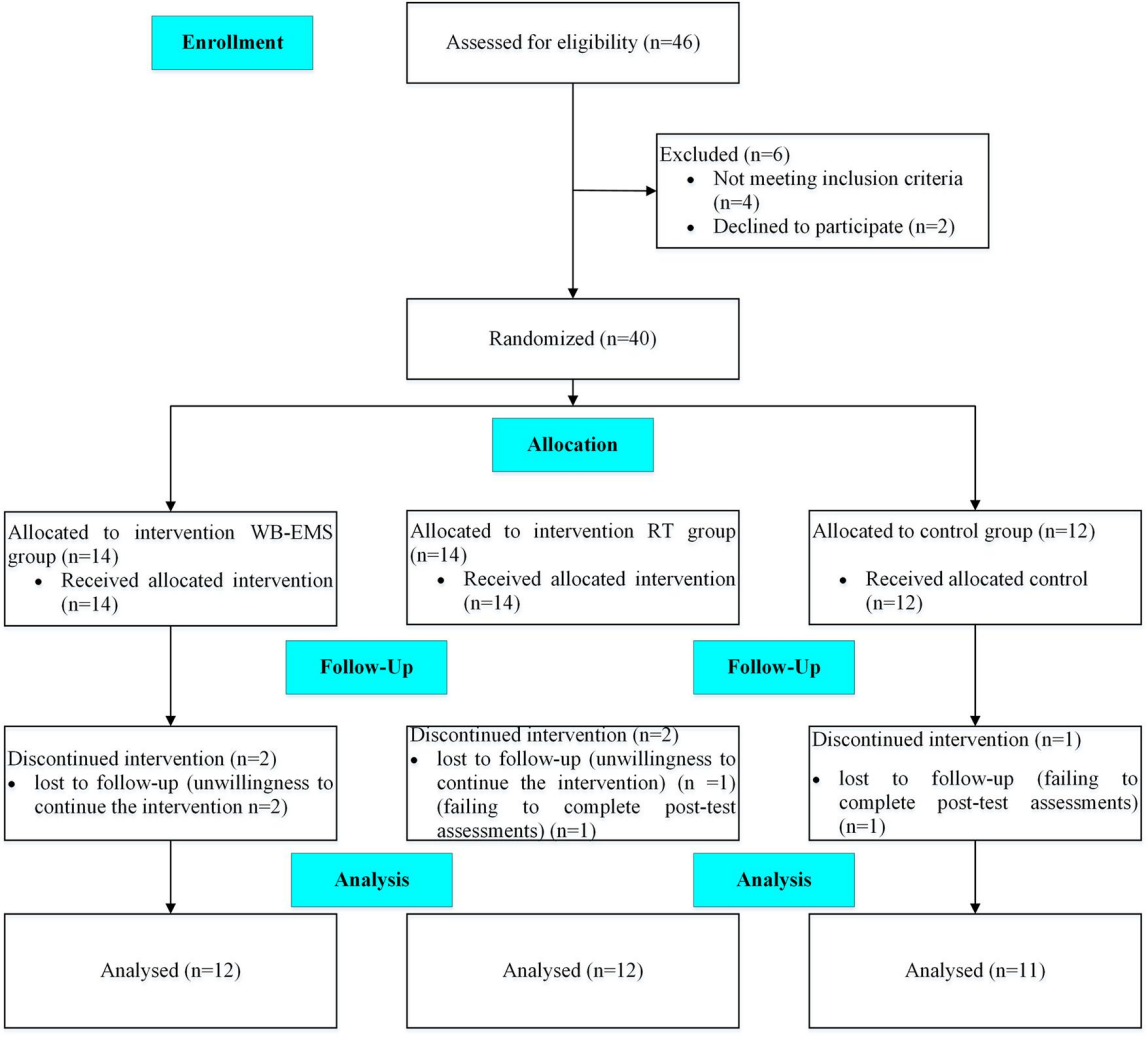
Flow diagram illustrating participant recruitment, randomization, allocation, follow-up, and analysis.

Overweight and obese adults aged 25–40 years were recruited from community health centers located in two districts of Tehran, Iran. A flow diagram illustrating participant recruitment, allocation, and follow-up is presented in Figure 1. Inclusion criteria included a body mass index (BMI) ≥ 25 kg/m^2^, absence of acute or chronic cardiovascular or orthopedic disorders, and no use of medications known to affect metabolic or physiological responses relevant to the study outcomes. Participants were excluded if they missed more than three consecutive training sessions, withdrew consent, or failed to complete post-intervention assessments.

Participants were instructed to maintain their habitual dietary patterns throughout the intervention period and to refrain from initiating any structured dietary programs. No formal dietary assessment (e.g., food records or 24-hour recalls) was conducted.

### 2.4 Training protocol

The 8-week experimental interventions comprised 16 supervised sessions (2 sessions per week), each structured in three standardized phases: a 5-minute dynamic warm-up, a 20-minute core training period, and a 5-minute stretching cool-down.

For the EMS group, participants performed dynamic movements while receiving whole-body electrical stimulation via the XBody PRO device (Gyor, Hungary). The stimulation protocol delivered biphasic electrical currents (30 Hz frequency, 350 μs pulse width) in intermittent cycles of 4-second stimulation intervals alternating with 6-second rest periods, totaling 20 min per session (28). The stimulation was administered through a multi-electrode vest targeting 10 major muscle groups (arms, back, chest, abdomen, thighs, glutes) with 18 contact points. To guarantee optimal conductivity, electrodes were hydrated with water before each session. Stimulation intensity was progressively titrated based on individual tolerance and recorded systematically to maintain treatment fidelity. The intensity was adjusted using the device's Master scale (0–10), which proportionally modulates the current delivered to all electrodes, allowing gradual increases based on each participant's tolerance and perceived contraction strength. All sessions were directly supervised by the principal investigator, who is an experienced EMS operator with full proficiency in device use, ensuring correct execution and safety. The training protocol incorporated bodyweight exercises, including squats, lunges, trunk flexions, lateral raises, biceps curls, and isometric abdominal contractions, all performed in a standing position without external loading. To enable voluntary muscle contractions during electrical stimulation, these movements were executed at a low intensity while standing.

The RT group completed a structured conventional resistance program comprising 20 min of core training preceded and followed by 5-minute warm-up and cool-down periods, respectively. The protocol incorporated three sets of 10 repetitions per exercise at 55%-70% of one-repetition maximum (1RM), with 30-second inter-set recovery intervals. Training intensity was progressively overloaded, initiated at 55% 1RM and increasing by 5% biweekly. Exercises targeted major muscle groups and included movements such as the chest press, leg press, shoulder press, squats, leg curls, bicep curls, lat pulldowns, and crunches. All sessions were conducted under the direct supervision of one of the investigators, an experienced certified strength and conditioning specialist, ensuring correct technique, protocol adherence, and participant safety.

Meanwhile, the control group (C) was instructed to maintain their habitual physical activity patterns and to refrain from initiating any structured exercise programs throughout the 8-week intervention. Although step counts were self-monitored using smartphone applications, step data were not formally recorded for statistical analysis. Weekly contact was maintained to reinforce adherence and minimize behavioral contamination. We acknowledge that objective monitoring of physical activity would have strengthened internal validity and this is now stated as a limitation.

Exercise intensity was quantitatively monitored using the Borg CR10 scale, which was administered immediately after each training session in both experimental groups. Participants rated their perceived exertion on a scale from 1 (“very, very light”) to 10 (“maximal exertion”) to provide a subjective assessment of session intensity (29).

Both intervention groups completed an identical frequency (two sessions per week), duration (8 weeks), and session length (∼20 min), resulting in comparable total training exposure time across modalities.

### 2.5 Study outcomes

Study outcomes were predefined prior to statistical analysis and categorized as primary and secondary endpoints.

Primary endpoint:

- Change in muscle thickness of the rectus abdominis and biceps brachii from baseline to 8-week follow-up, assessed by ultrasonography.

Secondary endpoints:

- Change in body fat percentage assessed by bioelectrical impedance analysis (InBody S10).
- Change in muscular performance measures, including abdominal muscular endurance (sit-up test) and back and leg strength (Takei dynamometer).
- Change in circulating myokine concentrations (interleukin-15, myostatin, follistatin, and F:M ratio).

All measurements in the pre-test and post-test were conducted by the same researcher between 9 and 11 AM in a university laboratory under standard ventilation, using similar devices.

#### 2.5.1 Anthropometric indices

Anthropometric measurements were obtained using calibrated instruments following standardized protocols. All anthropometric and body composition measurements were conducted by the same trained researcher in a laboratory between 9:00 and 11:00 AM to minimize diurnal variation. Participants were instructed to fast for prior to testing, avoid strenuous physical activity for 48 h, and refrain from alcohol and caffeine consumption for 24 h before assessment. They were also asked to maintain normal hydration and to empty their bladders immediately before measurement. Assessments were performed under standardized laboratory conditions with controlled ventilation and ambient temperature. All procedures followed the manufacturer's guidelines for the InBody device to ensure consistent electrode contact and standardized measurement conditions, thereby reducing potential measurement error and improving the reliability of BIA-derived outcomes. Height was measured to the nearest 0.1 cm using a wall-mounted stadiometer (Holtain Ltd., Crymych, UK). Body mass was assessed with a certified digital scale (Seca 769, Hamburg, Germany), with participants wearing light clothing. BMI was derived using the standard equation (mass[kg]/height[m]^2^). Body composition (body fat percentage) was assessed via direct-segmental, multi-frequency bioelectrical impedance analysis (DSM-BIA; InBody S10, Seoul, Korea).

Ultrasonographic measurements were performed using a high-resolution B-mode ultrasound system (Philips Clearview 550, Bothell, WA, USA) with a 7.5 MHz linear transducer. Muscle thickness of rectus abdominis (2 cm inferior to the xiphoid process) and biceps brachii (midpoint between humeral head and radial tuberosity) were measured. All scans were performed by a trained sonographer with participants in a supine position following a 15-minute resting period, applying minimal transducer pressure to avoid tissue compression. To ensure measurement consistency, all ultrasound assessments were performed by the same experienced operator using a standardized protocol, including identical anatomical landmarks, probe orientation, participant positioning, and device settings at both pre- and post-intervention assessments.

Although repeated within-session measurements were not performed and the same anatomical landmarks were marked at baseline to ensure consistency at post-intervention. In the present study to calculate study-specific reliability indices [e.g., coefficient of variation (CV) or intraclass correlation coefficient (ICC)], the reliability and validity of ultrasound-based muscle thickness measurements have been extensively documented in previous methodological studies. Prior research has reported excellent test–retest reliability, with ICC values typically ranging from 0.90 to 0.99 and CV values generally below 5% when standardized protocols are applied (30–32).

#### 2.5.2 Physical fitness assessments

Abdominal muscle endurance was assessed using a sit-up test. Participants lay supine on a mat with their knees flexed at a 90-degree angle and their hands crossed over their shoulders. They performed sit-ups by lifting their upper body until their elbows touched their knees, then lowered back to the starting position using their abdominal muscles. The total number of properly executed sit-ups within one minute was recorded (33).

To assess the strength of the back and leg muscles, a Takei back and leg dynamometer was used. Participants stood on the dynamometerˊs platform, gripping the handle with both hands. While maintaining a neutral spine, they extended their body upward to exert maximal force, and the peak value was recorded from the device display (34). Each participant performed the test three times, and the highest value was recorded.

#### 2.5.3 Blood sampling and analysis

Venous blood samples were collected from the antecubital vein between 8:00 and 8:30 AM, both before and after the intervention. Samples were centrifuged at 3,000 × g for 15 min to obtain serum, which was subsequently aliquoted and stored at −20 °C until analysis. Serum concentrations of interleukin-15 (IL-15; Cat. No. CYT-230), follistatin (Cat. No. RK00289), and myostatin (MSTN; Cat. No. RK01885) were determined using commercially available enzyme-linked immunosorbent assay (ELISA) kits (ZellBio GmbH, Germany) in accordance with the manufacturer's instructions.

### 2.6 Statistical analysis

The sample size was calculated *a priori* using G*Power software (version 3.1.9.7) (35). The statistical procedure was set to *F* tests with ANCOVA: fixed effects, main effects, and interactions. The following parameters were specified: effect size f = 0.6, *α* level = 0.05, statistical power (1−*β*) = 0.85, numerator df = 2, number of groups = 3, and number of covariates = 1. Under these assumptions, the required total sample size was 34 participants. The effect size (f = 0.6) was derived from pooled estimates reported in Rodrigues-Santana et al. (36). To account for potential attrition and feasibility considerations, 40 participants were recruited. Five participants discontinued participation after randomization and did not complete post-intervention assessments. As post-intervention outcome data were unavailable for these individuals, a full intention-to-treat analysis could not be performed. Therefore, statistical analyses were conducted using a per-protocol approach, including the 35 participants who completed both baseline and post-intervention assessments. Data are expressed as mean ± standard deviation (SD). Between-group differences in post-intervention outcomes were analyzed using one-way analysis of covariance (ANCOVA), with baseline values entered as covariates to control for initial variability. When significant main effects were observed, Bonferroni-adjusted *post hoc* comparisons were conducted to identify pairwise differences. Bonferroni-adjusted alpha was set at 0.0167 for pairwise comparisons (three comparisons). Effect sizes (ES) are reported as partial eta squared (*η*_p_^2^). Values of 0.01, 0.06, and 0.14 were interpreted as small, medium, and large effects, respectively. Statistical significance was set at *p* ≤ 0.05. All analyses were performed using IBM SPSS Statistics software (version 19.0; IBM Corp., Armonk, NY, USA). Figures were prepared in GraphPad Prism (Version 7.03, GraphPad Software).

## 3 Results

Forty eligible participants were enrolled and randomized. During the 8-week intervention, five participants withdrew (three due to unwillingness to continue and two due to failure to complete post-intervention assessments). Consequently, 35 participants (mean ± SD: age 32.2 ± 4.6 years; height 170.9 ± 9.5 cm) completed the study and were included in the final per-protocol analysis. All remaining participants in both groups completed the full 16 training sessions, and adherence to the prescribed exercise intensity was maintained throughout the intervention. No adverse effects were reported during the study.

Out of a total of 35 at baseline, 26 participants (74.3%) were classified as overweight and 9 participants (25.7%) as obese. Within the WB-EMS group, 9 participants (75.0%) were overweight and 3 (25.0%) were obese; in the RT group, 9 (75.0%) were overweight and 3 (25.0%) were obese; and in the C group, 8 (72.7%) were overweight and 3 (27.3%) were obese. There were no statistically significant differences in BMI classification between groups at baseline (*p* > 0.05).

### 3.1 Primary endpoint

#### 3.1.1 Muscle thickness

Significant between-group differences were observed in muscle thickness measurements, demonstrating a large effect size for the rectus abdominis (F₂, ₃₁= 56.24, *p* < 0.001, *η*_p_^2^ = 0.78) and a large effect size for the biceps brachii (F₂, ₃₁ = 28.67, *p* < 0.001, *η*_p_^2^ = 0.65) as summarized in Table 1. Bonferroni *post-hoc* analysis (adjusted alpha ≈ 0.0167) revealed statistically significant differences between both experimental groups and the control group (*p* < 0.01), as well as between the WB-EMS and RT groups specifically (*p* < 0.05).

**Table 1:** Anthropometric and performance parameters of participants pre- and post-intervention.

Variable	Group	Pre Mean ± SD	Post Mean ± SD	% change	*P*-value
Body mass (kg)	WB-EMS	82.14 ± 15.41	78.47 ± 14.42	−4.36	0.001
	RT	89.57 ± 15.09	88.80 ± 14.58*	−0.77	
	C	86.15 ± 15.95	86.72 ± 16.05*	0.67	
BMI (kg/m^2^)	WB-EMS	29.12 ± 3.29	27.85 ± 3.27	−4.36	0.001
	RT	29.14 ± 3.21	28.91 ± 3.13*	−0.77	
	C	29.17 ± 3.11	29.37 ± 3.19*	0.67	
Body fat percentage (%)	WB-EMS	29.97 ± 3.73	25.77 ± 4.25	−14.06	0.001
	RT	29.47 ± 3.11	28.62 ± 3.74 *	−3.09	
	C	27.38 ± 7.07	28.05 ± 7.31*	2.38	
Thickness of biceps brachii (mm)	WB-EMS	12.83 ± 1.97	15.73 ± 2.97	22.18	0.001
	RT	12.77 ± 2.92	14.46 ± 3.34 *	13.45	
	C	11.91 ± 2.02	11.91 ± 1.97 *#	0.11	
Thickness of rectus abdominis (mm)	WB-EMS	29.26 ± 5.08	31.46 ± 4.81	7.86	0.001
	RT	30.23 ± 3.28	30.89 ± 3.35*	2.19	
	C	25.64 ± 5.18	25.66 ± 5.21 *#	0.07	
Back and leg muscles strength (kg)	WB-EMS	162.08 ± 35.71	181.67 ± 35.69	12.99	0.001
	RT	182.92 ± 44.43	200.00 ± 42.10	10.49	
	C	179.09 ± 38.06	177.73 ± 36.42*#	−0.47	
Push-ups (n)	WB-EMS	24.92 ± 8.03	29.25 ± 8.17	20.08	0.001
	RT	22.42 ± 8.59	28.16 ± 9.14	28.42	
	C	27.36 ± 5.27	27.64 ± 5.76*#	0.79	
Sit-ups (n)	WB-EMS	28.25 ± 5.66	31.67 ± 6.54	12.34	0.001
	RT	34.08 ± 10.29	36.75 ± 9.91	9.22	
	C	28.64 ± 7.48	28.91 ± 6.42 *#	1.98	

*Significant difference with WB-EMS group, # significant difference with RT group.

WB-EMS, whole-body electromyostimulation; RT, resistance training; C, control;.

### 3.2 Secondary endpoints

#### 3.2.1 Anthropometric and performance indices

Baseline comparisons revealed no statistically significant variations among groups in anthropometric parameters (*p* > 0.05). Summary data for anthropometric and physical performance indices, measured before and after the intervention, are displayed in Table 1. ANCOVA results demonstrated statistically significant post-intervention differences between groups for body mass (*F*₂,₃₁ = 22.50, *p* < 0.001, *η*_p_^2^ = 0.59), BMI (*F*₂,₃₁ = 24.65, *p* < 0.001, *η*_p_^2^ = 0.61), and body fat percentage (*F*₂,₃₁ = 25.99, *p* < 0.001, *η*_p_^2^ = 0.63), all with large effect sizes. Bonferroni *post-hoc* analysis (adjusted alpha=0.05/3≈0.0167 for the three pairwise comparisons: WB-EMS vs. RT, WB-EMS vs. Control, RT vs. Control) indicated that the WB-EMS group differed significantly from the other groups in these three parameters.

As shown in Table 1, no significant between-group differences were observed in baseline physical performance tests (all *p* > 0.05). Post-intervention analyses revealed significant improvements with large effect sizes in sit-ups (*F*₂,₃₁ = 8.51, *p* = 0.001, *η*_p_^2^ = 0.35), leg and back strength (*F*₂,₃₁ = 13.20, *p* < 0.001, *η*_p_^2^ = 0.46), and push-ups (*F*₂,₃₁ = 18.14, *p* < 0.001, *η*_p_^2^ = 0.54). Bonferroni *post-hoc* tests (adjusted alpha ≈ 0.0167) indicated that both experimental groups showed significantly greater improvements in muscular endurance and strength outcomes compared to the control group (Table 1).

#### 3.2.2 Biochemical indicators

**Figure 2:**
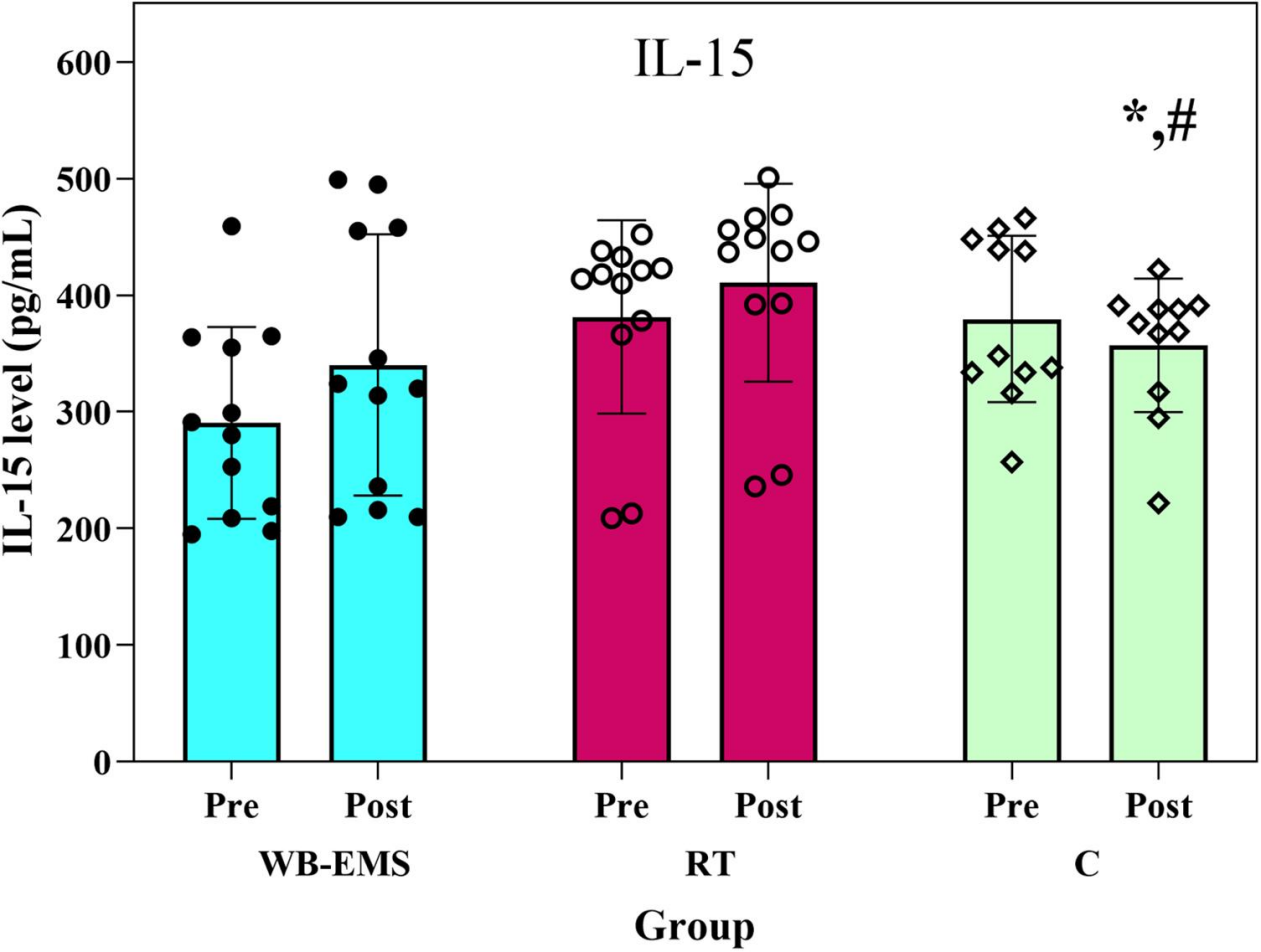
IL-15 level before and after the interventions. WB-EMS=whole-body electromyostimulation; RT = resistance training; C = control; *Significant difference with WB-EMS group, # significant difference with RT group.

ANCOVA revealed significant between-group differences in IL-15 levels post-intervention with large effect size (*F*₂,₃₁ = 9.00, *p* = 0.001, *η*_p_^2^ = 0.37). As shown in Figure 2, serum IL-15 concentrations increased significantly by 15.88% in the WB-EMS group and 8.27% in the RT group, while decreasing by 4.92% in the control group (Figure 2). *post-hoc* analyses demonstrated (adjusted alpha ≈ 0.0167) significant differences between both experimental groups and the control group (*p* < 0.001).

**Figure 3:**
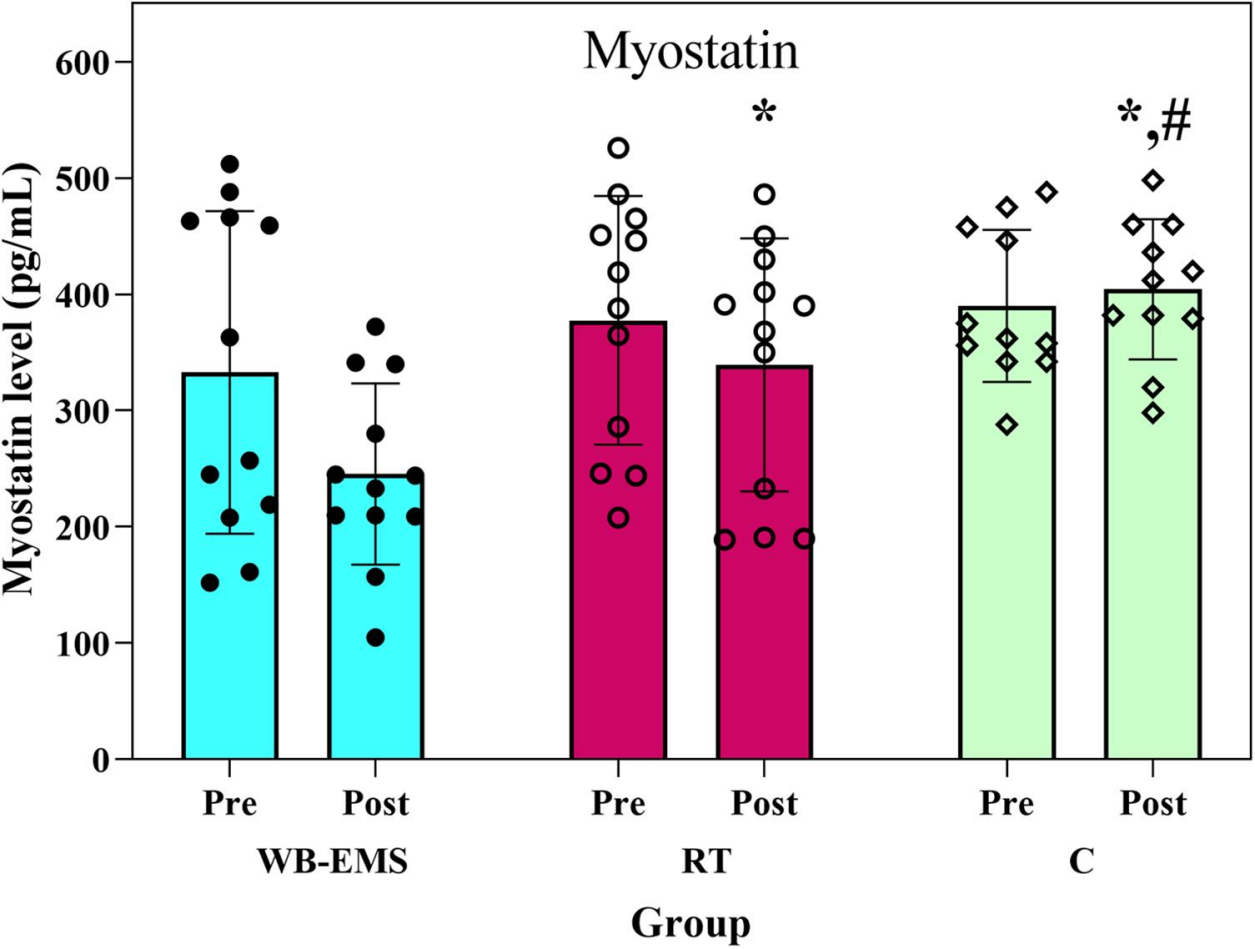
Myostatin level before and after the interventions. WB-EMS = whole-body electromyostimulation; RT = resistance training; C = control; *Significant difference with WB-EMS group, # significant difference with RT group.

Figure 3 shows the changes in serum MSTN levels in the study groups over the course of the intervention. ANCOVA revealed significant between-group differences in post-intervention MSTN levels with a large effect size (*F*₂,₃₁ = 14.79, *p* < 0.001, *η*_p_^2^ = 0.49). Serum MSTN decreased significantly by 21.22% in the WB-EMS group and 10.84% in the RT group, while increasing by 5.08% in the control group (Figure 3). Significant differences were observed between both experimental groups and the control group (*p* < 0.05), as well as between the WB-EMS and RT groups (*p* = 0.016).

**Figure 4:**
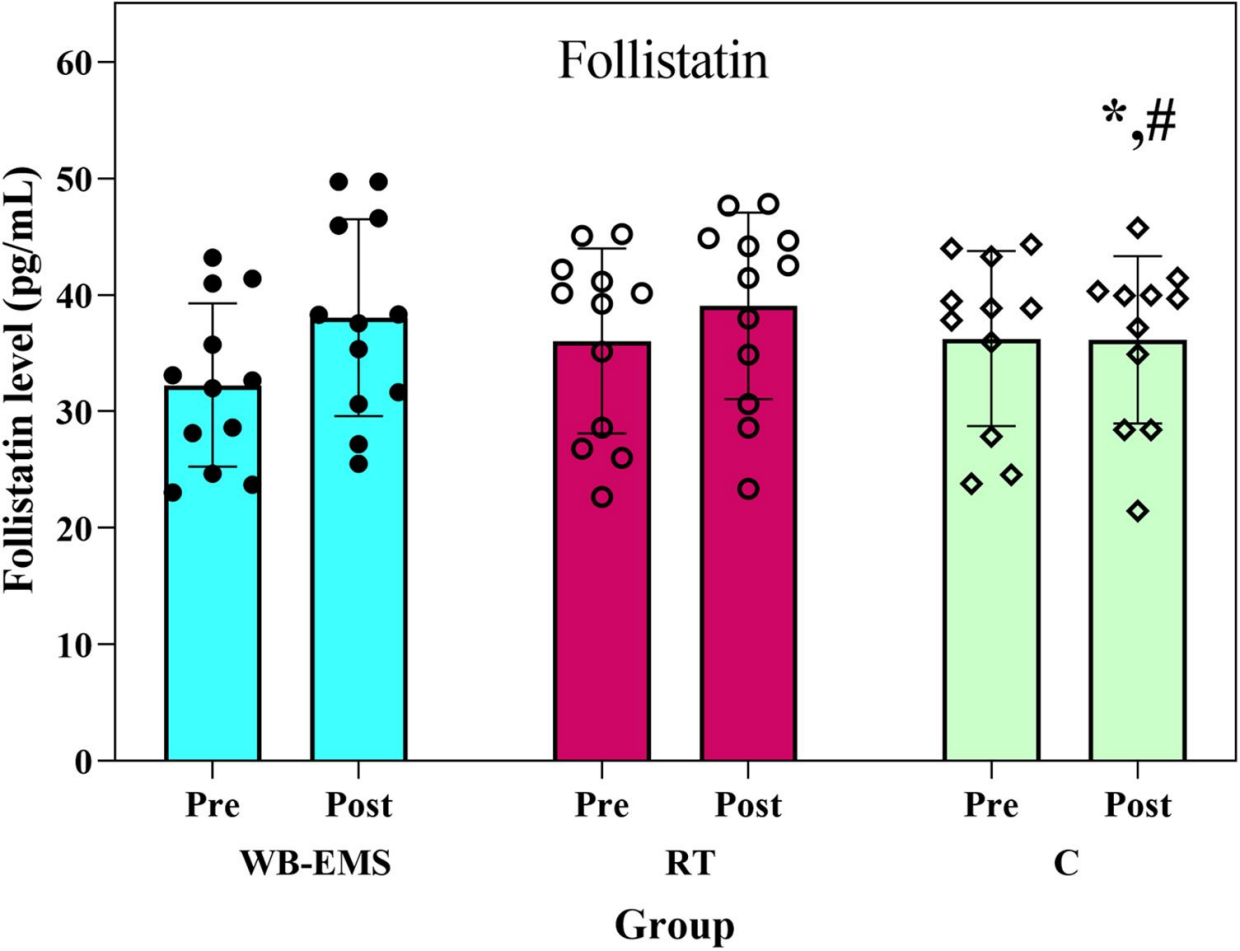
Follistatin level before and after the interventions. WB-EMS=whole-body electromyostimulation; RT = resistance training; C = control; *Significant difference with WB-EMS group, # significant difference with RT group.

Similarly, significant between-group differences were found in follistatin levels with large effect size (*F*₂,₃₁ = 8.32, *p* = 0.001, *η*_p_^2^ = 0.35). Serum follistatin increased by 10.02% in the WB-EMS group and 6.38% in the RT group, with minimal change (−0.10%) in the control group (Figure 4). Again, significant differences were detected between the control group and the WB-EMS (*p* < 0.001) and RT groups (*p* < 0.017). There was no significant difference between the two experimental groups (*p* = 0.059).

**Figure 5:**
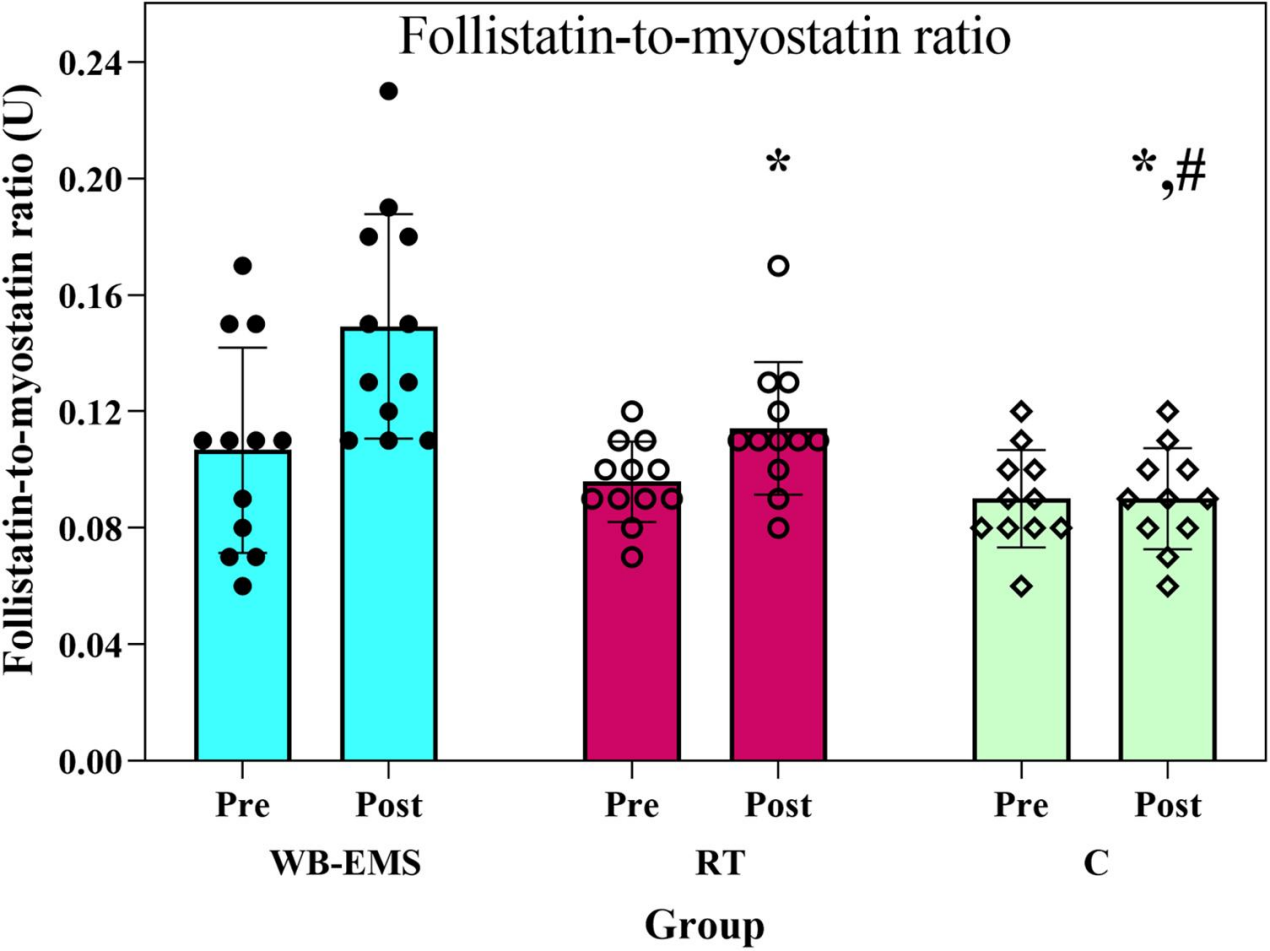
Follistatin-to-myostatin ratio before and after the interventions. WB-EMS=whole-body electromyostimulation; RT = resistance training; C = control; *Significant difference with WB-EMS group, # significant difference with RT group.

Statistical analysis revealed that the F:M ratio varied significantly among groups (*F*₂,₃₁ = 14.08, *p* < 0.001, *η*_p_^2^ = 0.48). The WB-EMS protocol resulted in a 48.9% improvement in this ratio (This relatively large percentage change should be interpreted in light of baseline variability), markedly surpassing the 20.8% gain in the RT group and the slight decrease observed in the control group (−2.9%), as illustrated in Figure 5. Corrected *post-hoc* testing (adjusted alpha ≈ 0.0167) confirmed that both active interventions differed from the control and that the WB-EMS group achieved a significantly higher response than RT (*p* = 0.028).

**Figure 6:**
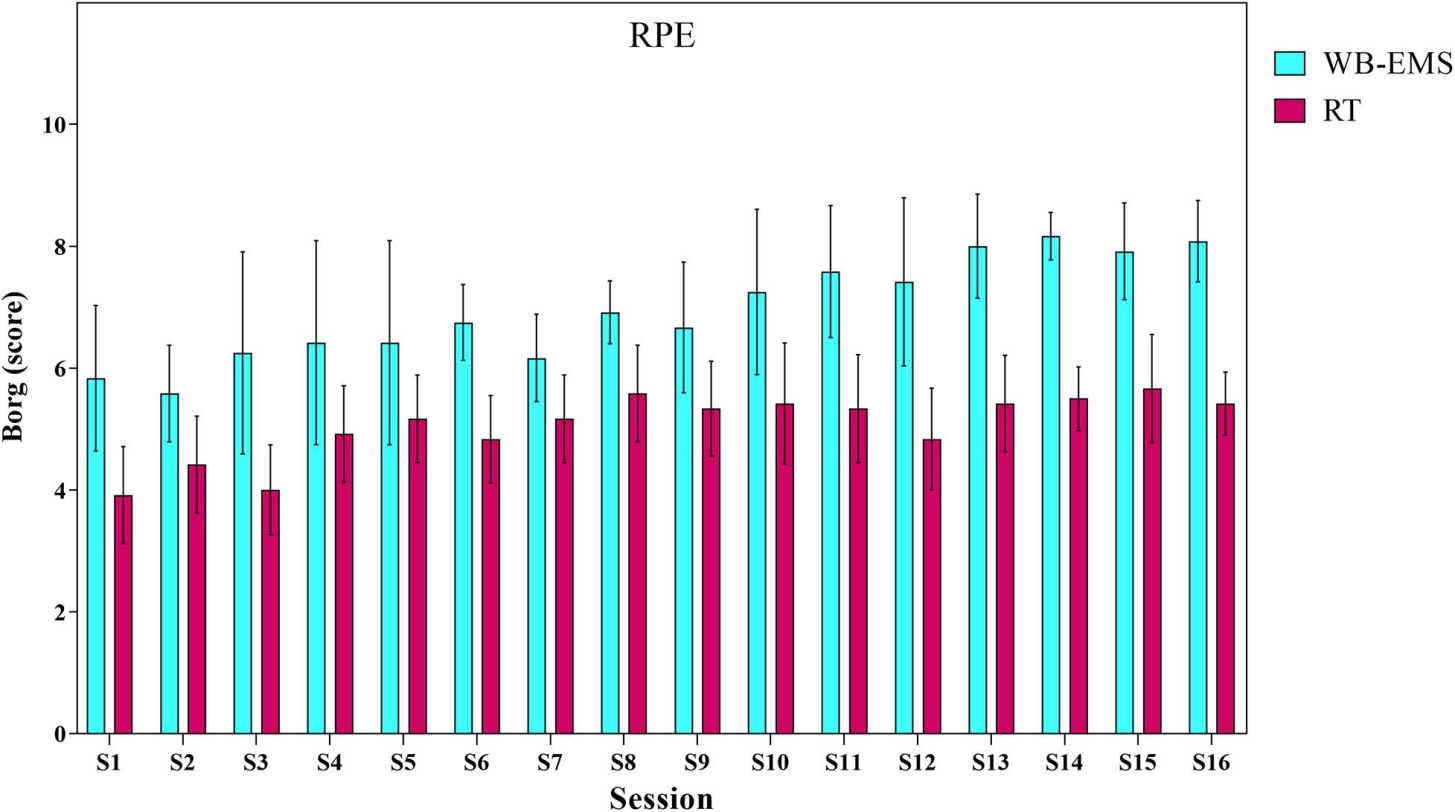
The rate of perceived exertion (RPE) for each group in each training sessions. RPE was greater in the WB-EMS group than in the RT group during all training sessions (*p* < 0.05).

As illustrated in Figure 6, from the first to the last week, significant differences in the rating of perceived exertion (RPE) were observed between the two groups, with RPE consistently higher in the WB-EMS group compared to the RT group.

## 4 Discussion

This study examined the comparative effects of 16 sessions of 20-minute WB-EMS vs. RT of equivalent duration on body fat percentage, BMI, muscle thickness of the rectus abdominis and biceps brachii, and myokine profiles. Prior to data collection, we hypothesized that both WB-EMS and RT would induce significant improvements compared with the control group, while potential differences between the two active interventions were explored without assuming superiority. WB-EMS tended to produce larger reductions in body fat percentage and more pronounced increases in muscle thickness (rectus abdominis and biceps brachii) compared to RT and the control group. Trends toward elevated IL-15 levels and improved F:M ratio were observed, accompanied by increased follistatin and decreased myostatin. These findings should be interpreted cautiously, considering potential influences of dietary intake, measurement variability (particularly in body composition), and other uncontrolled factors. Overall, the results suggest that WB-EMS could be a time-efficient modality for supporting muscular adaptations, but further studies are required to confirm its efficacy under controlled dietary conditions and with larger sample sizes.

Following the intervention, muscle thickness measurements revealed an increase in both training groups, with a more pronounced magnitude of hypertrophy observed in the WB-EMS group compared to the RT group. It is well-established that RT at 30%–90% of one-repetition maximum (1RM) induces muscle hypertrophy through mechanisms such as androgenic hormone release, satellite cell activation, and enhanced protein synthesis (37, 38). The extent of hypertrophy is known to depend on exercise volume and intensity (39). Notably, the WB-EMS group exhibited a greater degree of hypertrophy than the RT group. This difference may be attributed to the prolonged time under tension per muscle group during WB-EMS training compared to conventional RT. Such an extended muscular stimulation likely induces greater fatigue, thereby promoting more pronounced adaptive responses and subsequent hypertrophy. Consequently, the higher training volume in the WB-EMS group may account for its superior hypertrophic effects. Furthermore, increases in molecular markers associated with muscle hypertrophy—such as the F:M ratio and IL-15 concentration—were observed. These findings align with previous research; for instance, Sadeghipour et al. (2022) reported that WB-EMS enhances lean body mass (40). Similarly, Matos et al. (2022) documented significant increases in elbow flexor muscle thickness following eight weeks of RT, EMS, and combined training (41). The observed increase in muscle thickness justifies the improvements seen in performance indices. In summary, the present findings suggest that WB-EMS elicits greater muscle hypertrophy in the short term compared to traditional RT. However, further long-term investigations are warranted to validate these results.

Statistical analysis demonstrated a significant improvement in all three anthropometric indices—body weight, BMI, and body fat percentage—in the WB-EMS group compared with both the control and RT groups. Notably, the substantial reduction in body fat percentage observed in the WB-EMS group appears to account for the statistically significant differences in overall body composition measures. The pronounced decrease in fat mass may be attributed to enhanced metabolic activation and increased resting energy expenditure associated with WB-EMS (36, 42). WB-EMS elicits high levels of involuntary muscle fiber recruitment across multiple large muscle groups simultaneously, which may lead to elevated post-exercise energy expenditure and greater fat oxidation compared with conventional RT performed with similar duration and frequency (36, 42). Numerous factors can influence body composition, including dietary intake, underlying diseases, physical activity performed outside the study protocol, and medication use. Participants were monitored on a weekly basis throughout the study. Except for two participants who exhibited a reduction in body composition beyond the expected range—likely due to adherence to a more restrictive diet—the remaining participants maintained values within the normal range. Therefore, the observed changes can be primarily attributed to the training intervention.

An increase in circulating IL-15 levels was observed in both WB-EMS and RT groups compared to the control group. Acute exercise, regardless of modality, has been shown to elevate IL-15 levels within 10 min post-exercise (43, 44). Khalafi et al. (2024) further demonstrated in a systematic review that RT is more effective than aerobic exercise in modulating resting IL-15 levels, while combined training yields the largest effect sizes (45). These findings underscore IL-15's role as a contraction-sensitive myokine with both acute endocrine effects and chronic adaptive responses to training. As a multifunctional myokine, IL-15 possesses potent anti-obesity and anti-inflammatory properties, enhancing energy expenditure, promoting fat oxidation, reducing adipose tissue accumulation, and improving insulin sensitivity (46–48). Given that IL-15 secretion is stimulated by both metabolic and mechanical loading, the approximately two-fold greater increase in IL-15 levels observed in the WB-EMS group (15.9% vs. 8.3%) may be attributed to its broader muscle recruitment and higher metabolic demand compared to RT. The findings suggest that WB-EMS induces a more pronounced IL-15 response than RT, likely due to its greater metabolic and mechanical stimulus across multiple muscle groups. Given IL-15's critical role in metabolic regulation and muscle adaptation (47), these results highlight the potential of WB-EMS as an efficient strategy for enhancing myokine-mediated benefits.

The findings revealed that for the F:M ratio (increased follistatin, decreased myostatin), both interventions led to a marked increase compared to the control group, with the WB-EMS group exhibiting a twofold greater improvement than the RT group. Myostatin, a member of the TGF-*β* superfamily, is a potent inhibitor of muscle growth (49). In obesity, MSTN levels are often elevated, leading to muscle wasting, reduced energy expenditure, and worsened insulin resistance (2, 50). Six months of moderate aerobic exercise training significantly reduced MSTN levels in skeletal muscle and plasma, concomitant with improved insulin sensitivity (13). These findings support a causal role of MSTN in exercise-induced metabolic improvements. The systematic review by Khalafi et al. (2023) revealed that RT induces a significant decline in MSTN, accompanied by increased follistatin, resulting in an improved F:M ratio (51). This favorable modulation following RT may mechanistically contribute to the observed improvements in muscle hypertrophy and metabolic homeostasis. The current evidence indicates that WB-EMS significantly reduces serum MSTN concentrations (52). Exercise training downregulates MSTN through AMPK-mediated Smad2/3 inhibition (53) and JNK-mediated SMAD2 phosphorylation (54). In addition, increased follistatin induces muscle hypertrophy through direct binding and inhibition of MSTN (blocking its TGF-*β*/Smad signaling pathway) and inhibition of activin (55). Therefore, an elevated F:M ratio is associated with improved metabolic health and enhanced muscle mass.

The present findings should be interpreted within the context of several methodological limitations. First, participants were exclusively overweight/obese adults recruited from a single geographic region, which may limit the generalizability of the results to other populations. Second, the modest sample size (*n* = 35) may have constrained statistical power; although the selected effect size was informed by prior empirical findings, a more conservative medium effect size (f = 0.25) could have been selected, which would have required a larger sample size to achieve equivalent statistical power. This should be considered when interpreting the study's power assumptions. Third, the use of Bonferroni correction, while reducing the risk of type I error, may have further limited sensitivity to detect subtle between-group differences. Fourth, the lack of prospective trial registration represents an additional methodological limitation, as preregistration enhances transparency and reduces the risk of selective reporting bias. Fifth, a major limitation of the study is the absence of objective dietary assessment. Although participants were instructed to maintain their habitual dietary patterns and weekly reminders were provided to discourage intentional dietary modifications, no formal quantification of total energy intake or macronutrient composition was performed. Therefore, dietary stability cannot be confirmed. Unmeasured variations in nutritional intake may have influenced both body composition outcomes and circulating myokine responses and should be considered when interpreting the findings. Given the well-established interaction between dietary intake and changes in fat mass, lean mass, and metabolic signaling pathways, dietary factors should be considered a potential confounding variable. Furthermore, bioelectrical impedance analysis (BIA)-derived outcomes are inherently sensitive to hydration status and physiological fluctuations. Although standardized testing procedures were implemented to minimize variability, residual measurement error cannot be entirely excluded. Sixth, Phase angle, despite its relevance to cellular integrity and metabolic health, was not included among the predefined outcomes and was not archived in the exported dataset. Seventh, that within-session repeated ultrasound measurements were not performed to calculate study-specific reliability indices; however, all assessments were conducted by a single trained operator following previously validated procedures to reduce measurement variability. Additionally, the use of a per-protocol analytical approach following post-randomization attrition may limit internal validity and introduce potential attrition bias. Finally, the 8-week intervention period was sufficient to detect short-term adaptations but does not allow conclusions regarding the long-term sustainability of observed effects. Future randomized controlled trials should incorporate comprehensive dietary monitoring, preregistration, longer intervention durations, and expanded biomarker panels—including phase angle and additional myokines—to better isolate and understand the independent physiological effects of WB-EMS and resistance training.

WB-EMS may represent a time-efficient and joint-friendly alternative to traditional exercise, particularly beneficial for older adults and individuals with physical limitations. Evidence demonstrates its effectiveness in improving muscle strength, physical function, and myokine profiles (elevated IL-15 and F:M ratio) compared to RT. The technology's adaptability makes it suitable for those unable to perform vigorous exercise or with joint constraints. Given its dual benefits for body composition and molecular regulation of muscle metabolism, WB-EMS may be particularly valuable for combating obesity-related complications. Within the methodological limits of the present study, both WB-EMS and resistance training demonstrated beneficial adaptations compared with control, with some between-intervention differences that warrant further investigation in larger and longer-term trials.

## Data Availability

The original contributions presented in the study are included in the article, further inquiries can be directed to the corresponding authors.
